# Century-Long Warming Trends in the Upper Water Column of Lake Tanganyika

**DOI:** 10.1371/journal.pone.0132490

**Published:** 2015-07-06

**Authors:** Benjamin M. Kraemer, Simon Hook, Timo Huttula, Pekka Kotilainen, Catherine M. O’Reilly, Anu Peltonen, Pierre-Denis Plisnier, Jouko Sarvala, Rashid Tamatamah, Yvonne Vadeboncoeur, Bernhard Wehrli, Peter B. McIntyre

**Affiliations:** 1 Center for Limnology, University of Wisconsin-Madison, Madison, Wisconsin, United States of America; 2 NASA Jet Propulsion Laboratory, Pasadena, California, United States of America; 3 Finnish Environment Institute, Freshwater Centre, Jyväskylä, Finland; 4 Finnish Environment Institute, Marine Research Centre, Helsinki, Finland; 5 Department of Geography-Geology, Illinois State University, Normal, Illinois, United States of America; 6 Centre for Economic Development, Transport and the Environment for Pirkanmaa, Tampere, Finland; 7 Royal Museum for Central Africa, Tervuren, Belgium; 8 Department of Biology, University of Turku, Turku, Finland; 9 Department of Aquatic Sciences and Fisheries, University of Dar es Salaam, Dar es Salaam, Tanzania; 10 Department of Biological Sciences, Wright State University, Dayton, Ohio, United States of America; 11 Surface Waters Department, Swiss Federal Institute of Aquatic Science and Technology (Eawag), Kastanienbaum, Switzerland; 12 Institute of Biogeochemistry and Pollutant Dynamics, ETH Zurich, Zurich, Switzerland; The Ohio State University, UNITED STATES

## Abstract

Lake Tanganyika, the deepest and most voluminous lake in Africa, has warmed over the last century in response to climate change. Separate analyses of surface warming rates estimated from in situ instruments, satellites, and a paleolimnological temperature proxy (TEX_86_) disagree, leaving uncertainty about the thermal sensitivity of Lake Tanganyika to climate change. Here, we use a comprehensive database of in situ temperature data from the top 100 meters of the water column that span the lake’s seasonal range and lateral extent to demonstrate that long-term temperature trends in Lake Tanganyika depend strongly on depth, season, and latitude. The observed spatiotemporal variation in surface warming rates accounts for small differences between warming rate estimates from in situ instruments and satellite data. However, after accounting for spatiotemporal variation in temperature and warming rates, the TEX_86_ paleolimnological proxy yields lower surface temperatures (1.46 °C lower on average) and faster warming rates (by a factor of three) than in situ measurements. Based on the ecology of Thaumarchaeota (the microbes whose biomolecules are involved with generating the TEX_86_ proxy), we offer a reinterpretation of the TEX_86_ data from Lake Tanganyika as the temperature of the low-oxygen zone, rather than of the lake surface temperature as has been suggested previously. Our analyses provide a thorough accounting of spatiotemporal variation in warming rates, offering strong evidence that thermal and ecological shifts observed in this massive tropical lake over the last century are robust and in step with global climate change.

## Introduction

Climate change is altering the thermal characteristics of lakes worldwide, leading to a broad range of impacts on ecosystem processes [1]. Thermal characteristics of lakes directly influence water column stratification [2–4], water budgets, oxidation-reduction state [5], greenhouse gas efflux rates [6,7], and organismal metabolic rates [8]. Despite recognition that climate change has important direct and indirect effects on lake ecosystems, monitoring of long-term thermal changes in lakes remains limited.

Compared with temperate and arctic lakes, long-term, in situ lake temperature data sets are rare in the tropics. Satellite remote sensing of lake surface temperatures can redress the latitudinal bias of temperature monitoring for large lakes. Remote sensing typically yields comparable results to in situ monitoring [9,10], but is presently limited to only three decades of imagery. Conversely, paleolimnological temperature proxies can expand the temporal scales of lake temperature measurement, but the expense of core collection and analysis limits the spatial scope of this approach. Ideally, assessment programs should simultaneously consider multiple independent methods to cover longer timescales and allow maximal spatial and temporal resolution.

East Africa’s Lake Tanganyika has become one of the best-known cases of warming among the world’s lakes. As a result of the pattern of warming with depth, the lake has become more stratified, thereby reducing internal nutrient loading to the upper water column [11–13]. Evidence from sediment cores suggests that reduced internal nutrient loading has caused the lake to become less productive, with implications for the lake’s fishery [13,14] on which hundreds of thousands of people depend for their nutrition and livelihoods.

Multiple methods have independently been used to estimate the warming trends in this large (volume: 18,900 km^3^), old (~12 million years), and meromictic rift lake. The in situ water temperature record is one of the longest direct observation time series from any lake in the world. While historical temperature data are distributed across the entire spatial extent of the lake, previous analyses have focused on long-term warming trends in deep water (> 100 meters depth) in the north basin (~1/3 of the lake’s volume) in the wet season (October-April) where the data are the richest [11–13]. Lake surface temperatures have been measured using space-borne radiometers since 1985 including the Advanced Very High Resolution Radiometers (AVHRR) and the Along Track Scanning Radiometer (ATSR) [9]. The high-resolution satellite record (mean data gap is 5.1 days) complements the long-term but sporadic in situ temperature record from Lake Tanganyika. In addition to in situ instruments and satellites, the TEX_86_ paleotemperature proxy has been used to reconstruct 60,000 years of lake surface temperature data in Lake Tanganyika [14,15]. The TEX_86_ paleotemperature proxy uses the temperature dependence of Thaumarchaeal (planktonic microorganisms) glycerol dialkyl glycerol tetraether cyclization to reconstruct surface temperatures [16,17].

Published warming rate estimates from each method disagree by a factor of two. In situ instruments, the TEX_86_ paleolimnological proxy, and satellite-borne radiometers reporting warming rates of 0.15 [12], 0.21 [14], and 0.30°C decade^-1^ [9], respectively. This variation in temperature trend estimates could be attributable to spatiotemporal variation in warming rates because they were applied to different parts of the lake, different portions of the year, and different lengths of time. However, spatiotemporal variation in warming trends have not been explored in lake Tanganyika. The differences might also be attributable to inherent differences between the measurement approaches themselves (e.g. temperature at the air-water interface measured by satellites versus bulk surface temperatures measured by in situ thermometers). Our understanding of the impacts of warmer temperatures on Lake Tanganyika’s spectacular ecosystem would benefit from the reconciliation of all three perspectives to yield a more consistent estimate of warming rates.

In this paper, we assess whether warming rates in the upper water column (<100 m depth) vary spatially within the lake using in situ data and test whether spatiotemporal variation in temperature and warming rates can account for observed differences between measurement methods. To address these goals, we synthesized new and previously published in situ surface temperature data with other data sources (the TEX_86_ paleolimnological temperature proxy, the ATSR satellite instruments, and the AVHRR satellite instruments) for Lake Tanganyika. Using the in situ data, we develop models to characterize spatiotemporal variation in upper water column temperatures and rigorously compare model output to the other data sources. Our work demonstrates the need to explore spatial variation in the response of lake temperature to climate change within large lakes.

## Methods

### Study Location

**Fig 1:**
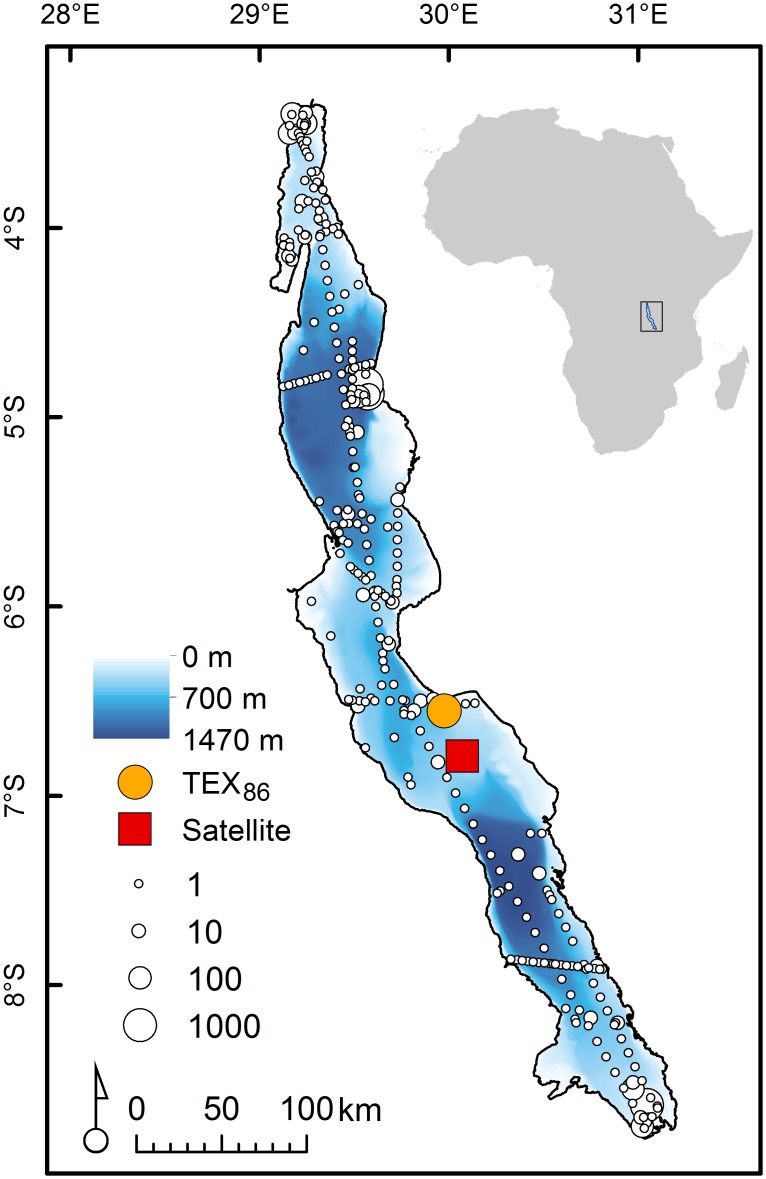
Map of Lake Tanganyika and its position in East Africa. White circles with black outlines indicate locations of in situ temperature measurement. The size of the circle is proportional to the number of temperature measurements taken at each location. Most of the in situ data come from areas near major research centers (Uvira, Kigoma, and Mpulungu). The orange circle indicates the location of the TEX_86_ sediment core and the red square indicates the location of the satellite data extraction site. The north, central, and south basins are located between 3.4–5.8°S, 5.8–7.0°S, and 7.0–8.9°S, respectively.

Lake Tanganyika is a long (650 km) and deep (1470 m) lake located in East Africa and oriented on a roughly north-south axis between 3.4 and 8.9°S latitude (Fig 1). It has three basins that are separated by relatively shallow transverse sills (~500 m depth). The north, central, and south basins are located between 3.4–5.8°S, 5.8–7.0°S, and 7.0–8.9°S, respectively. The south basin is the deepest while the north basin has the highest volume. Seasonal southeast trade winds during the dry windy season (May-October) and differential evaporative cooling over the 650 km length of the lake drives large scale convective circulation and internal waves with a period of 25 to 30 days [18]. The internal waves are reactivated at the end of the dry season (September) and persists through the rest of the year with decreased amplitude [18–20].

### Temperature Data

Several syntheses of in situ temperature data have been published for Lake Tanganyika [11–13]. These syntheses focused on deep water below 100 meters depth. Here we expand upon those records by including historical temperature data sets that were not included in previous work and by focusing on temperature in the upper water column (S1 Table). This enabled us to include data which span the spatial extent of the lake and the entire seasonal range. We also include new temperature data in the north, central, and south basins (S1 Table) that were collected near Kigoma, Mpulungu, and offshore from Tanzania’ Mahale Mountains National Park. Field research permits were granted by the Vice Chancellor of the University of Dar es Salaam. We collected these data between 1993–2013 using an YSI 6600 V2 data sonde, titanium RBRduo TD, Seacat Profiler V3.1b, and Onset HOBO U22 temperature loggers that were cross calibrated biannually. Temperature data taken before 1993 were measured using data loggers, standard mercury thermometers, and reversing thermometers. All temperature data taken over the last century reported here come from at least 1.5 km offshore and where the water depth exceeds at least 100 m. Much of the data come from sites near the three major research centers on the lake near Uvira, Democratic Republic of Congo; Kigoma, Tanzania; and Mpulungu, Zambia (Fig 1). In total, 13985 temperature observations were included in our analysis. The temperature data used here are freely available through the Long-Term Ecological Research network data portal [21] and as supporting information for this article (S4 Dataset in S1 Metadata).

TEX_86_ is a well-documented surface water temperature proxy applied to the open ocean and many large lakes that have well-preserved sediments with limited inputs of terrestrial organic matter [14]. Sediment cores from Lake Tanganyika were taken from the central basin of the lake at 6.552°S, 29.975°E (Fig 1) [14]. The original published TEX_86_ data [15] were recalibrated and updated in a subsequent publication [14]. The recalibration leads to a downward temperature correction of about 2.0°C. All data presented here are based on the updated calibration procedure. Uncertainty in the TEX_86_ temperature estimates arises from random error in the calibration and aging of the sediment core, but results are considered to be accurate to within 0.4°C for Tanganyika [14]. In total, 9 TEX_86_ temperature observations over the last century are included in our analysis, each interpreted as the mean annual surface temperature at the core site. The complete TEX_86_ temperature data used here are freely available through the World Data Center for Paleoclimatology [14].

Calibrated satellite lake skin temperature data for Lake Tanganyika spanning the seasonal range have been published in an online database for a single data extraction point in the central basin [9]. We update these published data with the addition of two years of data (2010–2011) processed by the same methods. The ATSR and AVHRR data used in this study were acquired for the periods when they were available from 1985–2011, and excluded pixels with cloud cover following the algorithms of previously published work [9]. Night-time ATSR and AVHRR data were extracted and averaged within a 3 X 3 km and 4 X 4 km area, respectively, centered over the location 6.792°S, 30.072°E in the central basin of the lake (Fig 1). Night-time data were used to avoid bias from orbital drift of the satellites. Satellite temperature estimates were calibrated and validated against buoy data from the Laurentian Great Lakes [9]. The mean and maximum data gaps for the Lake Tanganyika satellite data are 5.1 days and 112 days, respectively. The satellite data are freely available through the “Large Lakes” data portal on the National Aeronautics and Space Administration’s Jet Propulsion Laboratory website [9] and as supporting information for this article (S5 Dataset in S1 Metadata).

### Statistical modeling of in situ temperature

We developed a statistical model of in situ temperatures to (1) test whether upper water column (top 100 meters) warming rates vary spatiotemporally, and (2) determine whether differences in the location and time of satellite and TEX_86_ measurements can account for disparities in temperature and warming rates among these three methods. The spatial and temporal incompleteness of Lake Tanganyika’s in situ temperature data precluded traditional statistical modeling such as autoregressive integrated moving average (ARIMA) approaches. Instead, we build a series of general linear mixed effects models to all available temperature observations in the upper water column (≤ 100 m depth) made over the last century. We fit the model separately for data from 11 depths (0, 10, 20, 30, 40, 50, 60, 70, 80, 90, and 100) in the upper water column. The structure of the model reflects known and hypothesized drivers of temperature variation in the upper water column of Lake Tanganyika:

$$Y_{i,W}=\;L_{W}+\;S_{W}+\;\beta_{1}Decade+\;\beta_{2}GISS+\;\beta_{3}Decade*L+\beta_{4}Decade*S+\beta_{7}Decade*H_{W}+\;\epsilon_{i}$$

where *Y*_*i*,*w*_ is the *i*th temperature observation on the *w*th week of the year; *L*_*w*_ is the week of the year-specific effect of latitude (°S); *S*_*w*_ is the week of the year-specific effect of distance from shore (km); *Decade* is the decimal decade of each observation since 1900; *GISS* is the detrended Goddard Institute for Space Studies Land-Ocean Temperature Index (GISS) [22]; *L* and *S* are the latitude and distance to shore at the location of each measurement; *H*_*w*_ is the average relative percent humidity for the week of the year when the temperature measurement was made (averaged over a 2.5 year period from 2011–2013 from a weather station in Kigoma, Tanzania); and *ϵ*_*i*_ is the observation-specific error. In total, the models characterizes spatial, seasonal, interannual, and century-long variation in temperature.

The term in the model for latitude (*L*_*w*_) and distance to shore (*S*_*w*_) at the location of each measurement characterize spatial variation in temperature over the course of the year (but not inter-annual or century-long variation). *L*_*w*_ *and S*_*w*_ were fit separately for each week of the year because these effects are known to vary substantially within a season. In other words, *L*_*w*_, and *S*_*w*_ are interaction terms between week of the year (as a random effect) and two spatial predictors of temperature (*L*, *S*).

The *β* terms in the above equation are coefficients for the continuous, fixed effects in the model that characterize inter-annual and century-long variation in temperature. In part, this variation is captured by the first two fixed effects in the model; *Decade* and *GISS*. *β*_*1*_, the coefficient for the *Decade* term, can be interpreted as the generalized, century-long warming rate. *β*_*2*_, the coefficient for the *GISS* term, can be interpreted as the influence of global, inter-annual variation in temperature on upper water column temperatures in Lake Tanganyika. We include the detrended, monthly GISS index over the period from 1912–2013 as a predictor because inter-annual variation in Lake Tanganyika temperatures have been shown to closely track global air temperatures [12]. The GISS data have had the long-term trend removed so that the GISS coefficient (*β*_*2*_) describes the effect of interannual variation in global land surface temperatures but not the long-term warming trend.

The long-term warming trend has also been hypothesized to vary seasonally and spatially within the lake [14]. The continuous, fixed interaction terms in the model between *Decade* and latitude (*L*); *Decade* and distance from shore (*S*); and between *Decade* and the average relative humidity for week of the year (*H*_*w*_) characterize the influence of location within the lake and season on the long-term temperature trend. The average relative humidity on each week of the year was included in the model as an interaction term with *Decade* because humidity is tightly related to the multivariate seasonal axis from the cold, dry and windy season to the hot, wet, and less windy season. The *β* terms associated with these interaction terms (*β*_*3*_, *β*_*4*_, and *β*_*5*_ *)* can be interpreted as the impact of *L*, *S*, and *H* on the generalized warming rate estimate (*β*_*1*_).

To assess the accuracy of the model, we compare raw temperature measurements to modeled estimates using the root mean squared error (RMSE) from ordinary least squares regression. We used bootstrapping to minimize spatial and temporal autocorrelation in the temperature data; we randomly sample 10% of the temperature observations made over the last century for each 10 meter depth bin and fit the model to that subset of the data. We repeated this procedure 100 times with replacement and examined the distribution of each model parameter across models. By randomly subsetting the data across large spatial and temporal scales, we severely limit violations of the assumption that temperature data are not spatially or temporally autocorrelated. To interpret the robustness of our model, we compare the coefficients from the full model to the distribution of parameters derived from fitting data subsets. Because the median parameter is more robust to the violations of model assumptions, the median parameters from model subsets are used for predicting in situ temperature data at the sites of the satellite and TEX_86_ core extractions.

### *In situ* temperature comparison to TEX_86_ and satellites

The second major goal of the in situ temperature model was to determine whether the observed variation in warming rates based on in situ temperature data can account for differences in warming rate estimates across methods (in situ, satellites, TEX_86_). Ideally, TEX_86_ temperature data and satellite data could be compared directly to in situ temperature observations made in the same location. However, that is not possible with the available data. Instead, we used output from the in situ temperature model to predict surface temperatures at the satellite data extraction site and the TEX_86_ core site over time. First, we substituted the latitude (6.55°S) and distance from shore (5.62 km) at the location of the TEX_86_ core site into the temperature model fit to surface temperature data to generate daily estimates of surface temperature at the core site. We used the daily estimates of modeled, in situ surface temperature to calculate annual means for comparison to the raw TEX_86_ data. Similarly, we substituted the latitude (6.72°S) and distance from shore (30.69 km) at the location of the satellite extraction site into the temperature model to generate daily estimates of surface temperature at the site of the satellite extraction. The resulting model output is our best estimate of surface temperatures at those specific locations, and is informed by all available in situ data.

Temperature model output for the locations of the satellite extraction and the TEX_86_ core site were compared to raw data from each method using major axis (MA) regression. MA is a type II, least squares regression technique used when there is error in the measurements of both x and y variables [23]. The long-term warming rates associated with modeled in situ data, satellites and TEX_86_ were based on annual means. To estimate the surface warming rate based on TEX_86_ temperature data, we randomly resampled from the TEX_86_ error distribution (±0.4°C) [14] to account for uncertainty in the TEX_86_ calibration procedure. We used the resampled data to calculate the long-term trend. We repeated this resampling procedure 1000 times to estimate a distribution of warming rate estimates and an associated 95% confidence interval. Warming rates were compared using analysis of covariance (ANCOVA). All statistics were computed using R (R v3.1.0, core team, 2013).

## Results

### Statistical modeling of *in situ* temperature

The models fit to in situ temperature observations in the upper water column of Lake Tanganyika accounted for 44–89% of the variation in temperature depending on the depth to which the model was fit (S2 Fig). The fixed effects in the models which characterize inter-annual and century-long temperature trends explained progressively more of the temperature variation with increasing depth. The fixed effects explained 4% of the variation in surface temperature while they explained 45% at 100 m (S2 Fig). The RMSE of the temperature models decreased with depth from 1.19°C at the surface, to 0.21°C at 100 m (S2 Fig). Model residuals were normally distributed and unrelated to any of the predictors in the model.

The random effects of latitude (°S) and distance to shore (km) on temperature varied over the course of the year. Latitude showed strong seasonal variation in its impact on temperature. As expected, during the dry season, temperature increased with latitude and during the wet season temperature decreased with latitude. The distance from shore had a weak, seasonally variable impact on temperature; in the dry season, temperature tends to decrease with distance from shore and in the wet season temperature tends to increase with distance from shore. Surface temperatures were strongly related to the GISS-LOTI, but this relationship was less pronounced at depth and became slightly negative below 70 m (S3 Fig).

**Fig 2:**
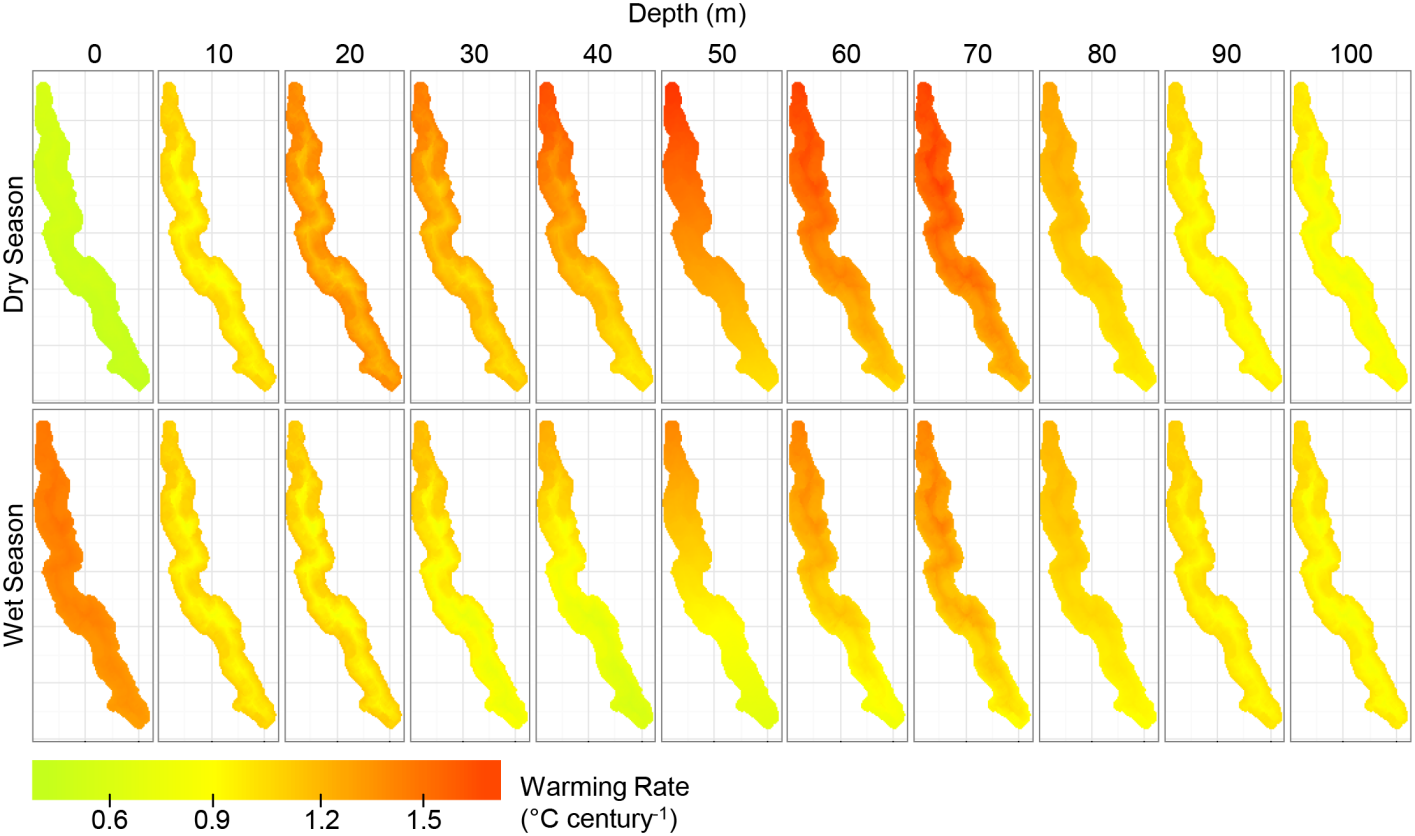
Modeled century-long warming rate estimates (1912–2013). Each colored pixel on the maps is an estimate of the warming rate for that location in the lake based on the temperature model fit to all available in situ temperature data. Separate map panels show variation across seasons and depths in the estimated warming rate. The top row of warming estimate maps are for the dry season and the bottom row of warming rates are for the wet season. The columns indicate the depth gradient of warming rate estimates from 0–100 m. All temperature models were fit to data from more than 1.5 km from land and in locations where the water depth exceeded 100 m deep.

According to the model of in situ temperatures, the surface of the lake has warmed on average at a rate of 0.129 ± 0.023°C decade^-1^ over the period 1912–2013. Water temperatures increased over the period 1912–2013 at all depths from 0–100 m across the spatial extent of the lake (Fig 2). The fastest warming rates on average can be found at depths of 50–80 m (Fig 2). The model results suggest that there is significant variation in warming rates over the surface of the lake and through the year. On average, warming rates at the nothern tip of Lake Tanganyika exceed warming rates at the southern tip by about 0.013°C decade^-1^ (Fig 2). This latitudinal difference is most pronounced at 50 m below the surface where the northern basin is warming 0.076°C decade^-1^ faster than the southern tip of the lake (Fig 2). The seasonal temperature cycle also influences the rate of temperature change. Surface warming rates vary by 0.080°C decade^-1^ over the seasonal cycle with the slowest surface warming rates occuring in the dry season when temperatures are typically lower (Fig 2). The opposite pattern (warming rate faster in dry season) is observed over the depth range from 20–80 m (Fig 2). The distance from shore also impacted the century-long warming rate but the effect was weaker than the effects of latitude and seasonality. The fastest surface warming rates occurred close to shore but for much of the water column (10–50 m and from 90–100 m), distance to shore was negatively related to warming rate (Fig 2).

Overall, the model coefficients for the full fitted model agreed well with the median coefficients from models fit to resampled 10% subsets of all data (S3 Fig). However, the full model tended to underestimate the effect of latitude and seasonality on warming rates, and overestimate the generalized warming rate and the effect of GISS-LOTI on temperature data (S3 Fig). The semi-parametric data subsetting approach is more robust than the full model which makes more assumptions about the underlying data. However, if the full model is more accurate, then the seasonal and latitudinal variation in warming rates estimated here may be overestimated.

### *In situ* temperature comparison to TEX_86_ and satellites

**Fig 3:**
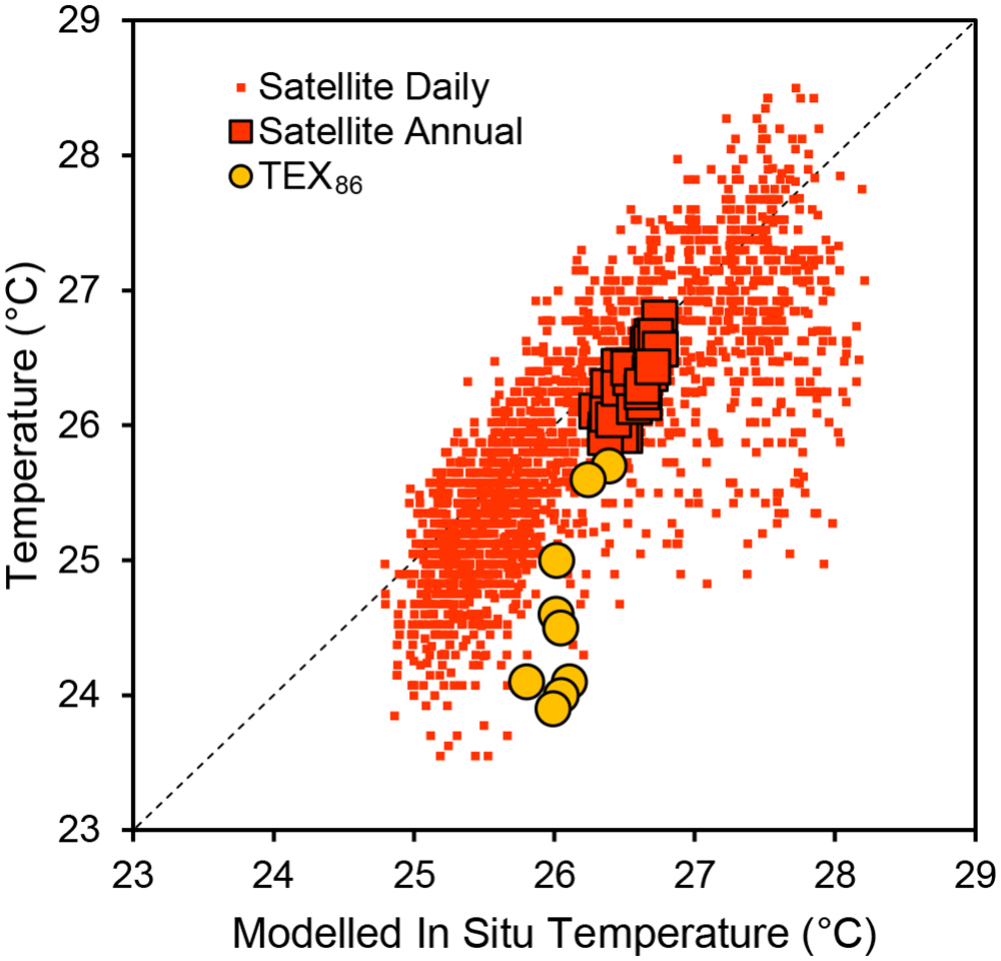
Satellite temperature and TEX_86_ temperature as a function of modeled in situ temperature. The black dashed line represents the 1:1 reference line. Small red square dots represent daily satellite temperatures as a function of the modeled in situ estimate at the satellite extraction site. Large red square dots with black outlines represent annual mean satellite temperatures as a function of modeled annual mean in situ temperatures at the extraction site. Annual mean satellite temperatures were calculated from raw satellite data linearly interpolated to daily timescales. Large orange circular dots with black outlines represent the TEX_86_ measurements as a function of the modelled annual mean temperature at the core site.

**Fig 4:**
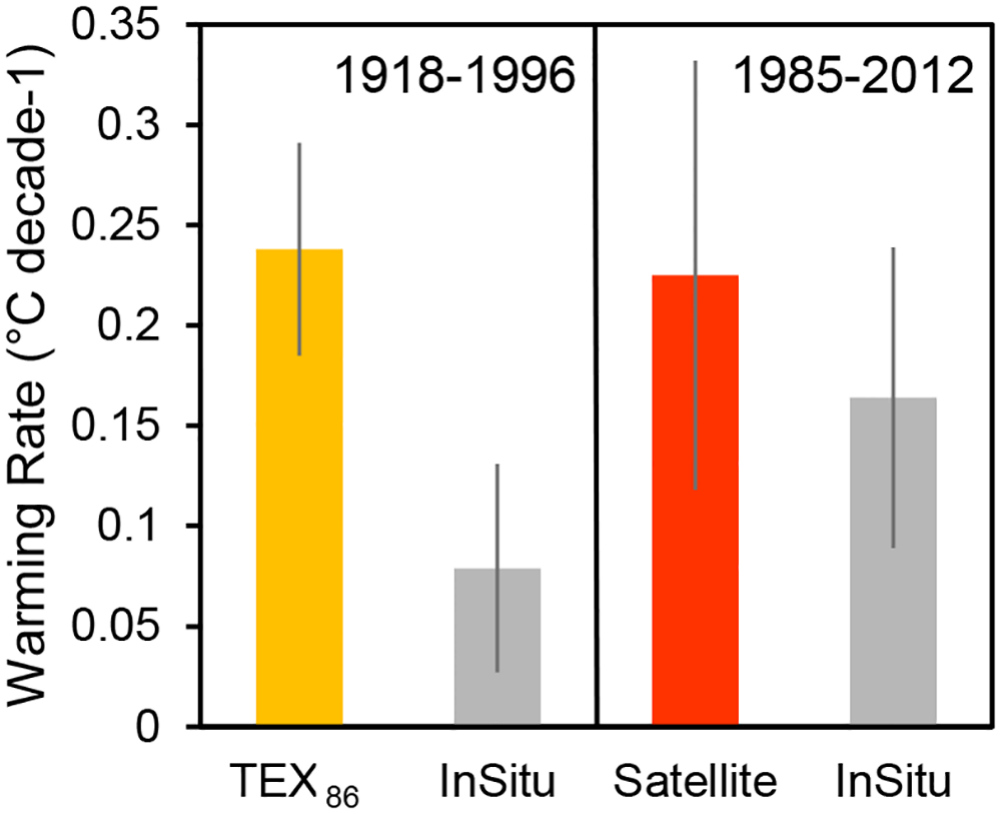
Surface warming rates based on in situ, satellite, and TEX_86_ data. Each bar represents a warming rate estimate based on one of the three temperature measurement methods (in situ, satellite, TEX_86_). Error bars represent 95% confidence intervals for each estimate. The two leftmost bars are warming rate estimates over the period from 1918–1996 based on TEX_86_ data and the in situ model output for the location and timeframe of the TEX_86_ core. The two rightmost bars show warming rate estimates over the period from 1985–2011 based on satellites and in situ model output for the location and time frame of the satellite data extraction.

**Fig 5:**
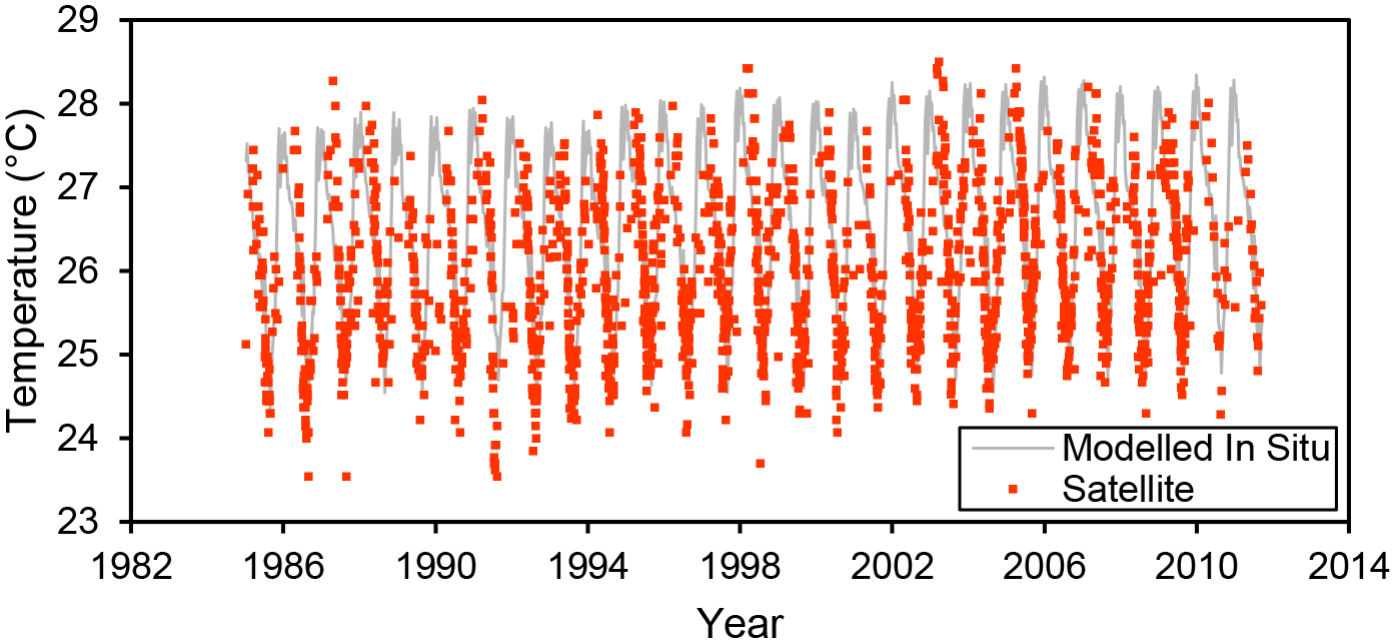
Long-term satellite temperature data compared to model estimates. Each small red square dot is a raw, satellite measurement of the lake’s surface. The grey line is the modeled surface temperature at the location of the satellite extraction. Satellite temperatures closely track the seasonal, interannual, and long-term variation in temperature data predicted by in situ temperature data.

Between 1985 and 2011, satellite temperatures were 0.26°C colder on average than the modeled in situ surface temperatures. Despite the difference in temperatures, there was no significant difference in surface warming rates between satellite data (0.225 ± 0.112°C decade ^-1^) and modelled in situ data (0.164 ± 0.075°C decade ^-1^) over the period from 1985–2011 (analysis of covariance, *p* = 0.26, Figs 3–5). The relationship between daily modeled in situ surface temperature (x axis) and satellite temperature (y axis) had a slope significantly greater than one (slope = 1.11, 95% confidence interval = 1.07–1.15, MA regression with 100 permutations, Fig 3). The slope of the annual averages of modeled in situ surface temperature (x axis) versus satellite temperature (y axis) was significantly greater than one (slope = 2.12, 95% confidence interval = 1.55–3.14, MA regression with 100 permutations, Fig 3).

**Fig 6:**
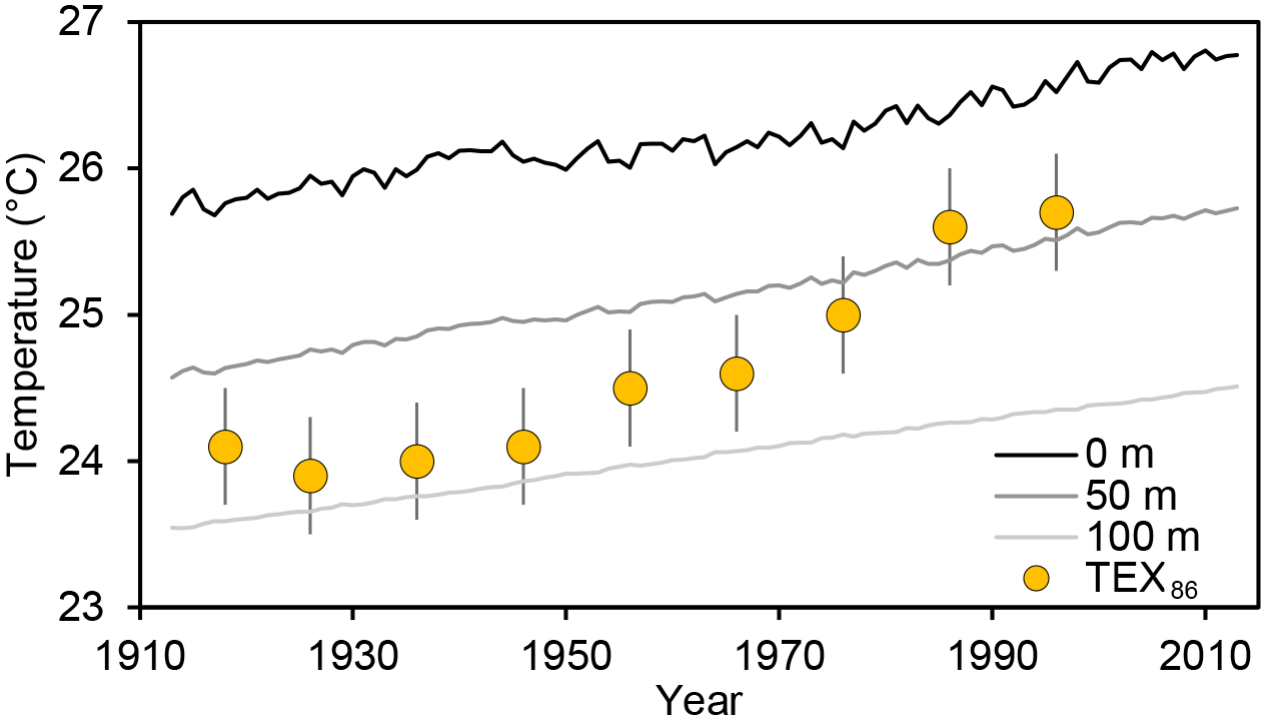
Long-term TEX_86_ temperature data compared to model estimates. Each large orange circular dot is a raw TEX_86_ surface temperature measurement. Each TEX_86_ measurement represents the annual mean surface temperature at the location of the TEX_86_ core site ± 0.4°C (95% confidence interval). The three lines are the modeled annual surface temperature at the location of the TEX_86_ core site at three different depths (0, 50, 100 m). There is a strong disagreement between TEX_86_ and modeled in situ temperatures, especially early in the time series. Deviations between the TEX_86_ temperature measurements and the modeled surface temperature may reflect model error, error in the TEX_86_ calibration procedure, or long-term shifts in the depth range of Thaumarchaeota (the microbes whose biomolecules are involved with generating the TEX_86_ proxy).

TEX_86_-based temperatures were 1.46°C colder on average than the modeled in situ surface temperatures (Fig 3). TEX_86_-based warming rates were faster than in situ temperatures by a factor of 3 over the period from 1918 to 1996 (0.248 ± 0.053°C decade ^-1^ for TEX_86_ versus 0.079 ± 0.052°C decade ^-1^ for modeled in situ data, Fig 4). After accounting for uncertainty in the TEX_86_ temperature calibration using monte carlo simulations with 100 permutations, there was still a significant difference in warming rates between TEX_86_ and the modeled in situ temperature trend (analysis of covariance *p* < 0.01, Figs 4 and 6). The slope between annual modeled in situ surface temperature (x axis) and TEX_86_ temperature (y axis) was significantly greater than one (slope = 5.29, 95% confidence interval = 2.96–20.91, MA regression with 100 permutations, Fig 4).

## Discussion

All three methods (in situ, satellite, TEX_86_) suggest that the surface of Lake Tanganyika warmed significantly over the last century (linear regression, *p*<0.05, Figs 4–6). Our analyses provide a detailed portrait of spatiotemporal patterns of warming in the upper water column of Lake Tanganyika, revealing that previous work on long-term warming in deeper water capture only a portion of the changes over the last century. Spatial variation in warming rates partially account for the differences between warming rate estimates based on in situ, satellite and TEX_86_ data. However, even after accounting for spatiotemporal variation in temperature and warming trends, key differences among methods remain.

Spatiotemporal variation in warming rates appears to be linked to vertical mixing patterns within the lake. Warming rates tend to be slower in areas of the lake with relatively high vertical mixing. For instance, warming rates are slower in the southern latitudes where vertical mixing is greater. The most persistent vertical mixing in the southern basin occurs in the dry windy season when wind-induced tilting of the thermocline induces ‘primary upwelling’ as the thermocline is lifted toward the surface there. Primary upwelling may slow apparent surface warming rates by transporting accumulated surface heat to deeper water and refreshing the surface with cold hypolimnetic water annually. Thus, in effect surface heat is distributed into a much larger mass of water than the local epilimnion, yielding a slower warming rate in the upper water column. Enhanced vertical mixing has also been suggested as a mechanism for slower warming in the upper water column at the onset of summer in Lake Ontario [24]. Areas of Lake Ontario that were more strongly stratified warmed faster at the surface than areas with lower stratification [24]. A similar pattern was observed in Lake Tanganyika but over interannual timescales instead of over seasonal timescales.

Seasonal differences in the pattern of warming with depth also suggest that warming rates are linked to patterns of vertical mixing. Surface warming rates in the wet season were faster than warming rates from 10–100 m whereas surface warming in the dry season is slower than at depth. This pattern can be explained by seasonal differences in the strength of microstratification at the surface of the lake. Due to weakening of surface winds in the wet season, a secondary thermocline typically forms in the top 5–15 meters of the water column in Lake Tanganyika [25]. The secondary thermocline serves as a barrier to mixing and may prevent the transfer of heat to deeper depths in the wet season. Heat that gets trapped in the top 5–15 m of the water column above the secondary thermocline may be lost back to the atmosphere through outgoing long wave radiation, sensible heat loss, or latent heat loss. However, latent heat losses may be relatively small in the wet season due to high humidity and low wind speeds. In the dry season, persistent trade winds disrupt the secondary thermocline, thus heat may be transferred to greater depths at that time of year. Thus seasonal variation in surface microstratification may explain season differences in the pattern of warming with depth.

Periodic, localized vertical mixing also occurs in near shore areas of Lake Tanganyika when internal waves interact with the lake bottom [26]. Turbulent mixing of this sort is likely to transmit heat from the surface to deeper parts of the lake, thereby slowing apparent surface warming rates near shore. The coefficient in our model associated with the interaction term between distance to shore and *Decade* suggested that warming rates may be slower nearshore only at the surface and from 60–80 m depth. Thus our model does not strongly support the hypothesis that warming rates closer to shore at the surface are slower than warming rates over deeper water where internal waves interact less strongly with the lake bottom. We may not have detected a strong effect of distance from shore on warming rates because we excluded data that were taken from areas shallower than 100 m and less than 1.5 km from shore.

The in situ temperature model suggests that the northern basin of the lake is warmer on average and experiencing relatively fast surface warming rates. This result contrasts with the global latitudinal gradient in lake warming where temperature is negatively correlated to warming rates [9]. The warmest time of year in Lake Tanganyika (wet season) has the fastest surface warming rates. At the global scale, warming is often slower at times of the year when temperatures are high [27], which also contrasts with our finding that water temperatures in the warm, wet season are warming faster.

### *In situ* temperature comparison to satellites

The difference between satellite and modelled in situ temperatures is at least partly attributable to differences in the time of day when satellite and in situ data are collected. In situ temperature data are measured during daylight hours when surface temperatures are at or near daily peaks, whereas satellite data are measured at night to avoid bias from orbital drift of the sensors [28]. At night, surface temperature is typically 0.2–0.6°C lower than the daytime temperature [25,29]. Thus, the average observed temperature difference between satellites and in situ instruments (0.26°C lower for satellites) could be entirely explained by the timing of observations. Furthermore, the difference between nightime and daytime surface temperatures are lowest in the cooler dry season when winds disrupt daytime surface microstratification. Similarly, the difference between satellite (night) and in situ (day) temperatures are also smallest in the cooler dry season. This further supports the hypothesis that differences between in situ and satellite-based surface temperature estimates primarily reflect differences in the time of day when temperature is measured by the two methods.

Even after accounting for diurnal variation in surface temperatures, there remains a substantial amount of unexplained variation in the comparison between daily satellite and daily in situ temperatures (Fig 3). Some of this variation may arise from spatial or temporal biases in satellite data collection. For instance, non-random spatial variation in surface temperature and atmospheric interference (clouds, smoke) could bias satellite temperature measurements [30]. Additionally, satellite data represent only cloud-free days, which could bias the annual mean surface temperature estimates toward warmer temperatures. Differences between temperature at the air-water interface (skin temperatures) and bulk surface water temperature may also explain adiditional variation in the difference between satellite and modeled in situ temperature. For example, satellite-based temperatures often exceed in situ measurements by ~0.2°C in lakes due to the differences in heat exchange between the atmosphere and skin water versus surface bulk water [10,30,31]. While inherent differences in the measurement approaches may be reflected in our data, satellite data and in situ data match relatively closely (Figs 3 and 4) and the differences between satellite and in situ temperatures observed here likely reflect the time of day when measurements are made.

### *In situ* temperature comparison to TEX_86_

The substantial differences between TEX_86_ temperature data and the modeled in situ temperature data at the location of the sediment core raise questions about interpretation of TEX_86_ data. Based on the pattern of mismatch between in situ bulk temperature and TEX_86_ temperature (Fig 6), it appears that the TEX_86_ paleolimnological proxy substantially and consistently underestimates the surface temperature in Lake Tanganyika using the current TEX_86_ calibration method. Given the direction of the mismatch, the TEX_86_ record may be most closely related to temperature at a fixed depth below the surface, or the average temperature over a range of depths [32]. The quantitative agreement between TEX_86_ temperatures and in situ temperatures over the last century is closest for in situ measurements taken at ~60 m depth where temperatures are colder than the surface. However, warming rates inferred from TEX_86_ are still much greater than those at 60 m according to our in situ temperature model for that depth. Furthermore, the satellite-based temperature data were extracted from images near the site where the sediment core was collected for TEX_86_ analysis (Fig 1), yet the satellite-based warming rate is similar to modelled in situ data and far lower than TEX_86_ temperatures. Together, these comparisons suggest that it is unlikely that warming at the core site was truly 3 times faster than that indicated by in situ data.

We propose an alternative explanation for the mismatch between TEX_86_ and in situ temperature data that is rooted in the ecology of Thaumarchaeota. Though no single abiotic shift is sufficient to explain why TEX_86_ suggests more rapid warming than in situ data in Lake Tanganyika, the observed shallowing of the oxycline suggest that TEX_86_ could reflect a biological response overlaid upon the in situ warming pattern. In Lake Tanganyika, Thaumarchaeota are most productive in the zone of the lake with moderate to low oxygen levels (“suboxic zone,” 0.5–4.0 mg L^-1^ dissolved oxygen, 40–180 m depth) [32]. The suboxic zone has shallowed over the last century due to reduced vertical mixing [11–13,33], suggesting that Thaumarchaeota have likely moved into shallower, warmer water. The magnitude of this inferred shift in the depth of Thaumarchaeota is comparable to the upward shift observed in the depth niche of endemic deep water molluscs in response to the shallowing oxic zone in Lake Tanganyika [33]. Shallowing of Thaumarchaeota’s oxygen niche would lead to warmer TEX_86_ temperatures over time irrespective of warming rates at any specific depth. Thus, TEX_86_ could be responding to an ecological change that is indirectly linked to the temperature that it purportedly measures [16,34].

The combination of climate-mediated warming at all depths with upward shifts in the oxygen niche of Thaumarchaeota is sufficient to explain the observed differences in both temperatures and warming rates between methods. If this interpretation is correct, the TEX_86_ paleolimnological proxy is still valid but may be more closely related to the temperature in the suboxic zone, not temperature at the surface or any other fixed depth. As a measure of suboxic zone temperature, the TEX_86_ record augments previous analyses of shallowing trends in snail depth distributions, and directly connects the measured temperature increases and estimated stabilization of the water column with organismal responses to climate change in Lake Tanganyika. Thus, our reinterpretation of the TEX_86_ record could lead to a more nuanced view of the entire 60,000 year temperature time series derived from TEX_86_ data for Lake Tanganyika. Without accounting for this ecological perspective, the most rapid rates of warming and cooling in the TEX_86_ record may be especially exaggerated, and published temperatures are likely to reflect temperature at a particular depth only during periods of relative stasis in suboxic zone depth.

## Conclusions

Our update and synthesis of in situ temperature data from Lake Tanganyika demonstrates that long-term warming rates in Lake Tanganyika vary with latitude, distance from shore, and with the seasonal cycle. Though our statistical approach to the incomplete space-time matrix of in situ observations from Lake Tanganyika has limitations, it nonetheless reveals consistent patterns of spatial variation in warming that illustrate a need to assess the spatial dimensions of warming within large lakes. This variation is most likely driven by variation in vertical mixing; areas and times of year with low vertical mixing experience the fastest warming rates. This pattern may be observed in other large lakes with spatial and temporal variation in vertical mixing patternsed quantitativeterpretationof water.odelled in situ temperature revealed important differences between thee methods and a. There are few large lakes where time series of in situ observations encompass a wide enough range of locations to estimate spatial variation in warming rates. Such comparisons have been made over shorter time scales in other large lakes, and have revealed substantial differences in warming across the surface of lakes [35,36]. The analyses of spatial variation in lake skin temperatures for Lake Tanganyika from satellites could be an informative complement to our work on spatially distributed in situ data.

The broad quantitative agreement between temperature records from satellites and in situ instrumental data engenders new confidence in the records themselves as well as their implications for climate change effects. Unfortunately, there are few lakes in the world that have temperature records from multiple methods spanning over a century of change. Of the lakes that have well-preserved sediments, few also have both long-term in situ temperature records and surface area large enough for unobstructed satellite-based measurements. Lakes Baikal and Malawi could provide all three types of records, thereby broadening perspectives on the effects of climate change on surface temperatures as well as testing whether our reinterpretation of the TEX_86_ paleolimnological temperature proxy could be correct for other lakes.

As the oxic zone shallows in Lake Tanganyika, aerobic organisms will be forced upward in the water column where temperatures are both warmest already and rising fastest. Higher temperatures exact metabolic costs for these organisms, potentially reducing their discretionary energy available for growth and reproduction [37,38]. Reduced vertical mixing associated with thermal shifts has already diminished internal nutrient loading, leading to reduced primary productivity and shifts in phytoplankton assemblages [11,13,14]. Warm lakes like Lake Tanganyika are especially vulnerable to warming-driven shifts in lake stratification due to the nonlinear relationship between water density and water temperature. The combination of reduced oxic habitat, increased metabolic demands, and lower primary productivity is likely to have negative effects on the lake’s ecosystem and the hundreds of thousands of people who depend on the lake for their nutrition and livelihoods.

## Supporting Information

S1 Dataset In situ temperature data. (TXT)

S2 Dataset Satellite temperature data. (TXT)

S1 Fig Mixed effects model evaluation. Root mean squared error (RMSE) and coefficient of multiple determination (R^2^) for mixed effects models fit to in situ temperature data as a function of depth. The variance explained by the fixed effects in each model is reported as “R^2^ Fixed Effects.”
(PDF)

S2 Fig Fixed effects model parameters. The *β* terms in the figures are coefficients for the continuous, fixed effects in the in situ temperature models. These models characterize inter-annual and century-long variation in Lake Tanganyika temperature as a function of depth. *β*_*1*_, the coefficient for the *Decade* term, can be interpreted as the century-long warming rate for a specific depth (°C decade^-1^). *β*_*2*_, the coefficient for the *GISS* term, can be interpreted as the influence of global, inter-annual variation in temperature on upper water column temperatures in Lake Tanganyika. The *β* terms associated with the interaction terms in the model (*β*_*3*_, *β*_*4*_, and *β*_*5*_ *)* can be interpreted as the impact of latitude (°S), relative humidity, and distance to shore (km) on the generalized warming rate estimate (*β*_*1*_). Blue dots indicate the median coefficient estimate across all models fit to 10% subsets of temperature data. Error bars extending from the blue dots represent the standard deviation in model coefficient estimates across models fit to data subsets. The empty circles represent coefficients from the full models fit to all available temperature data at a specific depth.
(PDF)

S1 Metadata Metadata for supplemetary datasets S4 and S5. (CSV)

S1 Table In situ temperature data sources. All in situ data sources for temperature data included in the temperature data synthesis. Season is reported as either dry (May-October) or wet season (November-April). Basin is reported as north, central, and south basins located between 3.4–5.8°S, 5.8–7.0°S, and 7.0–8.9°S, respectively. For each year-season-basin data combination, the original source of the data is reported as well as whether or not data have been included in previous long-term trend estimation. The vast majority of year-season-basin combinations have not been included in previous long-term temperature trend estimation. In total, temperature data are now available from the years 1912–3 [39,40], 1938–9 [41], 1946–7 [42], 1953 [42],1955–7 [43], 1960–2 [44], 1964–6 [44], 1973 [45], 1975 [46], 1981–2 [47], and intermittently from 1991 through 2013 [11,13,19,20,25,48–50]. A portion of the data from some of these sources has been used in previous analyses, but our inclusion of all three basins and both wet and dry seasons enabled us to use additional temperature data that was excluded from previous syntheses [39–42,44]. We also included data in our analyses from published sources that have never before been used in long-term temperature syntheses [26,47,49]. When raw data were not directly available, temperature data were digitized from figures in publications.
(DOCX)
