# Correction: Century-Long Warming Trends in the Upper Water Column of Lake Tanganyika

**DOI:** 10.1371/journal.pone.0134537

**Published:** 2015-07-30

**Authors:** The PLOS ONE Staff

There is an error in the fourth sentence of the first paragraph of the Conclusions section. The correct sentence is: This pattern may be observed in other large lakes with spatial and temporal variation in vertical mixing patterns. There are few large lakes where time series of in situ observations encompass a wide enough range of locations to estimate spatial variation in warming rates.

## References

[1] Kraemer BM, Hook S, Huttula T, Kotilainen P, O’Reilly CM, Peltonen A, et al (2015) Century-Long Warming Trends in the Upper Water Column of Lake Tanganyika. PLoS ONE 10(7): e0132490 doi: 10.1371/journal.pone.0132490 26147964 10.1371/journal.pone.0132490 PMC4492510

