# Supplementary figures and images for: Century-Long Warming Trends in the Upper Water Column of Lake Tanganyika
Source: PLoS One. 2015 Jul 6;10(7):e0132490. doi: 10.1371/journal.pone.0132490 (PMC4492510; doi:10.1371/journal.pone.0132490)

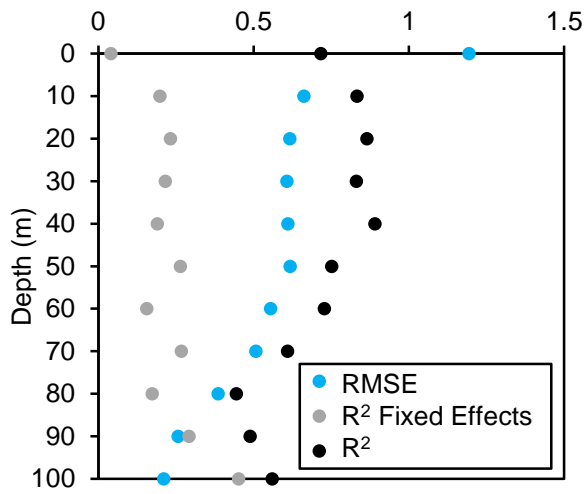

**S2 Figure**

Supplement: S1 Fig — Root mean squared error (RMSE) and coefficient of multiple determination (R2) for mixed effects models fit to in situ temperature data as a function of depth. The variance explained by the fixed effects in each model is reported as “R2 Fixed Effects.” (PDF) [file pone.0132490.s003.pdf]

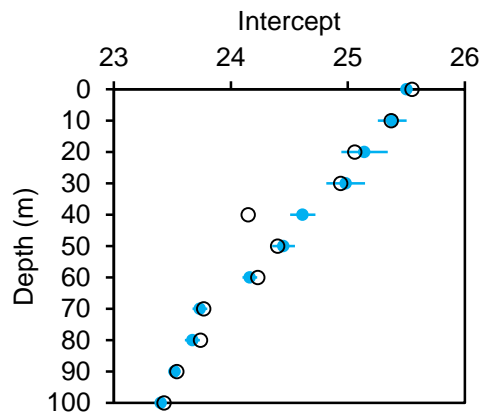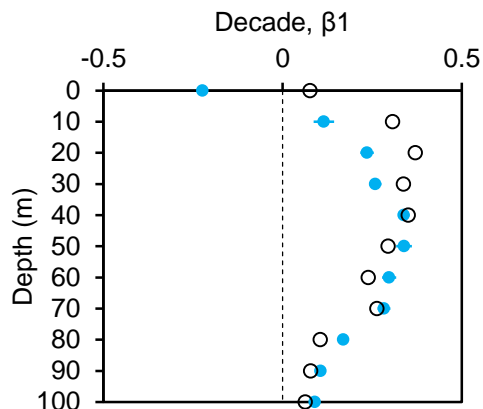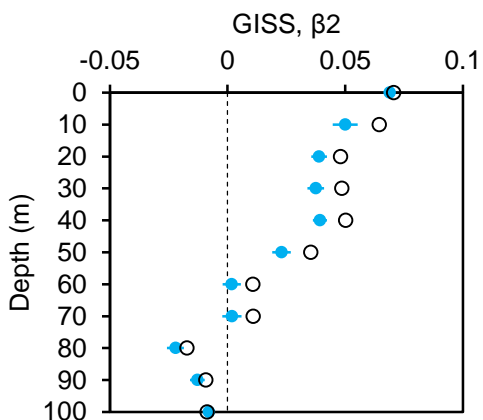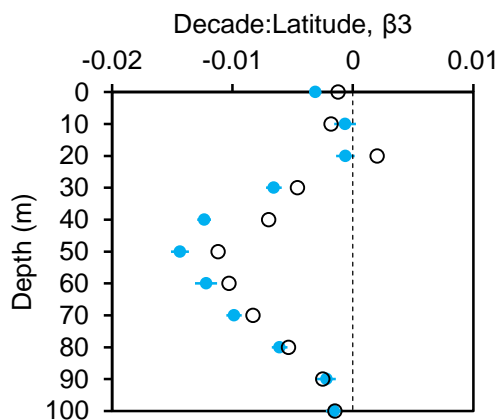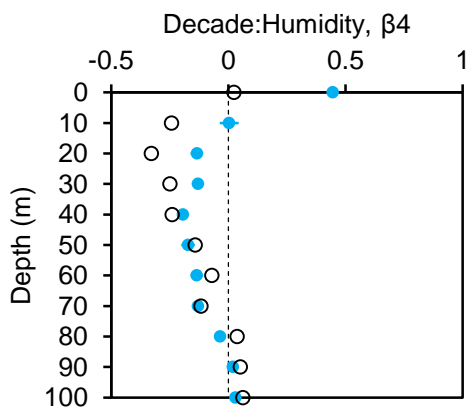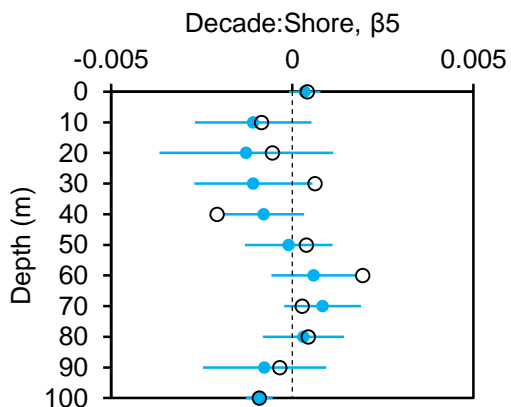

**S3 Figure**

Supplement: S2 Fig — The β terms in the figures are coefficients for the continuous, fixed effects in the in situ temperature models. These models characterize inter-annual and century-long variation in Lake Tanganyika temperature as a function of depth. β 1, the coefficient for the Decade term, can be interpreted as the century-long warming rate for a specific depth (°C decade-1). β 2, the coefficient for the GISS term, can be interpreted as the influence of global, inter-annual variation in temperature on upper water column temperatures in Lake Tanganyika. The β terms associated with the interaction terms in the model (β 3, β 4, and β 5 ) can be interpreted as the impact of latitude (°S), relative humidity, and distance to shore (km) on the generalized warming rate estimate (β 1). Blue dots indicate the median coefficient estimate across all models fit to 10% subsets of temperature data. Error bars extending from the blue dots represent the standard deviation in model coefficient estimates across models fit to data subsets. The empty circles represent coefficients from the full models fit to all available temperature data at a specific depth. (PDF) [file pone.0132490.s004.pdf]
