# Supplementary material for: Century-Long Warming Trends in the Upper Water Column of Lake Tanganyika
Source: PLoS One. 2015 Jul 6;10(7):e0132490. doi: 10.1371/journal.pone.0132490 (PMC4492510; doi:10.1371/journal.pone.0132490)
Supplement: S1 Table — All in situ data sources for temperature data included in the temperature data synthesis. Season is reported as either dry (May-October) or wet season (November-April). Basin is reported as north, central, and south basins located between 3.4–5.8°S, 5.8–7.0°S, and 7.0–8.9°S, respectively. For each year-season-basin data combination, the original source of the data is reported as well as whether or not data have been included in previous long-term trend estimation. The vast majority of year-season-basin combinations have not been included in previous long-term temperature trend estimation. In total, temperature data are now available from the years 1912–3 [39,40], 1938–9 [41], 1946–7 [42], 1953 [42],1955–7 [43], 1960–2 [44], 1964–6 [44], 1973 [45], 1975 [46], 1981–2 [47], and intermittently from 1991 through 2013 [11,13,19,20,25,48–50]. A portion of the data from some of these sources has been used in previous analyses, but our inclusion of all three basins and both wet and dry seasons enabled us to use additional temperature data that was excluded from previous syntheses [39–42,44]. We also included data in our analyses from published sources that have never before been used in long-term temperature syntheses [26,47,49]. When raw data were not directly available, temperature data were digitized from figures in publications. (DOCX) [file pone.0132490.s006.docx]

| S3 Table: In situ temperature data sources | | | |  |
| --- | --- | --- | --- | --- |
| Year | Season | Basin | Data source | Previous Trend Estimates |
| 1912 | Dry | North | [40] | None |
| 1912 | Dry | South | [40] | None |
| 1912 | Wet | Central | [40] | None |
| 1912 | Wet | North | [40] | None |
| 1912 | Wet | South | [40] | None |
| 1913 | Dry | Central | [39] | None |
| 1913 | Dry | North | [39] | [11,12] |
| 1913 | Dry | South | [39] | None |
| 1913 | Wet | Central | [40] | None |
| 1913 | Wet | North | [40] | [11–13] |
| 1913 | Wet | South | [40] | None |
| 1938 | Dry | Central | [41] | None |
| 1938 | Dry | North | [41] | [13] |
| 1938 | Dry | South | [41] | None |
| 1938 | Wet | North | [41] | None |
| 1939 | Wet | North | [41] | None |
| 1946 | Dry | Central | [42] | None |
| 1946 | Dry | North | [42] | [13] |
| 1947 | Dry | Central | [42] | None |
| 1947 | Dry | North | [42] | None |
| 1947 | Wet | Central | [42] | None |
| 1947 | Wet | North | [42] | [12] |
| 1947 | Wet | South | [42] | None |
| 1953 | Dry | North | [42] | None |
| 1953 | Wet | North | [42] | [13] |
| 1955 | Wet | North | [43] | None |
| 1956 | Dry | North | [43] | None |
| 1956 | Wet | North | [43] | None |
| 1957 | Wet | North | [43] | None |
| 1960 | Dry | South | [44] | None |
| 1960 | Wet | South | [44] | None |
| 1961 | Dry | South | [44] | None |
| 1961 | Wet | South | [44] | None |
| 1962 | Wet | South | [44] | None |
| 1964 | Dry | Central | [44] | None |
| 1964 | Dry | North | [44] | None |
| 1964 | Dry | South | [44] | None |
| 1965 | Wet | Central | [44] | None |
| 1965 | Wet | North | [44] | None |
| 1965 | Wet | South | [44] | None |
| 1966 | Wet | South | [44] | None |
| 1973 | Wet | North | [45] | [11–13] |
| 1975 | Wet | North | [46] | [11–13] |
| 1975 | Wet | South | [46] | None |
| 1981 | Dry | Central | [47] | None |
| 1981 | Wet | Central | [47] | None |
| 1981 | Wet | North | [47] | None |
| 1982 | Dry | North | [47] | None |
| 1982 | Wet | North | [47] | None |
| 1991 | Dry | North | [20,48] | None |
| 1992 | Wet | North | [20,48] | None |
| 1993 | Dry | North | [20,48] | None |
| 1993 | Dry | South | [4] | None |
| 1993 | Dry | South | [20,48] | None |
| 1993 | Wet | North | [20,48] | None |
| 1993 | Wet | North | [13] | [13] |
| 1993 | Wet | South | [4] | None |
| 1993 | Wet | South | [20,48] | None |
| 1994 | Dry | North | [20,48] | None |
| 1994 | Dry | South | [4] | None |
| 1994 | Dry | South | [20,48] | None |
| 1994 | Wet | North | [20,48] | None |
| 1994 | Wet | North | [13] | [13] |
| 1994 | Wet | South | [4] | None |
| 1994 | Wet | South | [20,48] | None |
| 1995 | Dry | North | [20,48] | None |
| 1995 | Dry | South | [4] | None |
| 1995 | Dry | South | [20,48] | None |
| 1995 | Wet | North | [20,48] | None |
| 1995 | Wet | North | [13] | [13] |
| 1995 | Wet | South | [4] | None |
| 1995 | Wet | South | [20,48] | None |
| 1996 | Dry | North | [20,48] | None |
| 1996 | Dry | South | [4] | None |
| 1996 | Dry | South | [20,48] | None |
| 1996 | Wet | North | [20,48] | None |
| 1996 | Wet | South | [4] | None |
| 1996 | Wet | South | [20,48] | None |
| 1997 | Wet | South | [4] | None |
| 1998 | Dry | North | [50] | None |
| 1998 | Dry | North | Previously unpublished | None |
| 1998 | Wet | North | [50] | None |
| 1999 | Dry | North | [50] | None |
| 1999 | Dry | North | Previously unpublished | None |
| 1999 | Wet | North | [50] | None |
| 2000 | Dry | Central | Previously unpublished | None |
| 2000 | Dry | North | Previously unpublished | None |
| 2000 | Dry | North | Previously unpublished | None |
| 2000 | Dry | North | [11–13] | [11–13] |
| 2001 | Dry | Central | Previously unpublished | None |
| 2001 | Dry | Central | Previously unpublished | None |
| 2001 | Dry | North | [49] | None |
| 2001 | Dry | North | Previously unpublished | None |
| 2001 | Dry | North | [13] | [13] |
| 2002 | Dry | Central | Previously unpublished | None |
| 2002 | Dry | North | [49] | None |
| 2002 | Dry | North | Previously unpublished | None |
| 2002 | Dry | North | [13] | [13] |
| 2002 | Dry | South | [49] | None |
| 2002 | Wet | North | Previously unpublished | None |
| 2003 | Dry | Central | Previously unpublished | None |
| 2003 | Dry | North | [49] | None |
| 2003 | Dry | North | Previously unpublished | None |
| 2003 | Dry | South | [49] | None |
| 2003 | Wet | North | Previously unpublished | None |
| 2003 | Wet | North | [13] | [13] |
| 2004 | Dry | North | [26] | None |
| 2004 | Wet | Central | Previously unpublished | None |
| 2004 | Wet | North | [49] | None |
| 2004 | Wet | North | Previously unpublished | None |
| 2004 | Wet | South | [49] | None |
| 2005 | Dry | North | Previously unpublished | None |
| 2005 | Wet | North | Previously unpublished | None |
| 2006 | Dry | North | Previously unpublished | None |
| 2006 | Wet | North | Previously unpublished | None |
| 2007 | Dry | North | Previously unpublished | None |
| 2010 | Dry | North | Previously unpublished | None |
| 2011 | Dry | North | Previously unpublished | None |
| 2011 | Wet | North | Previously unpublished | None |
| 2012 | Dry | Central | Previously unpublished | None |
| 2012 | Dry | North | Previously unpublished | None |
| 2012 | Wet | North | Previously unpublished | None |
